# Cancer risk in *MLH1*, *MSH2 *and *MSH6 *mutation carriers; different risk profiles may influence clinical management

**DOI:** 10.1186/1897-4287-7-17

**Published:** 2009-12-23

**Authors:** Dewkoemar Ramsoekh, Anja Wagner, Monique E van Leerdam, Dennis Dooijes, Carli MJ Tops, Ewout W Steyerberg, Ernst J Kuipers

**Affiliations:** 1 Department of Gastroenterology and Hepatology Erasmus MC University Medical Center, PO Box 2040, 3000 CA, Rotterdam, the Netherlands; 2 Department of Public Health, Erasmus MC University Medical Center, PO Box 2040, 3000 CA, Rotterdam, the Netherlands; 3 Department of Clinical Genetics, Erasmus MC University Medical Center, PO Box 2040, 3000 CA, Rotterdam, the Netherlands; 4 Department of Human and Clinical Genetics, Leiden University Medical Center, PO Box 9600, 2300 RC Leiden, the Netherlands; 5 Department of Internal Medicine, Erasmus MC University Medical Center, PO Box 2040, 3000 CA, Rotterdam, the Netherlands

## Abstract

**Background:**

Lynch syndrome (LS) is associated with a high risk for colorectal cancer (CRC) and extracolonic malignancies, such as endometrial carcinoma (EC). The risk is dependent of the affected mismatch repair gene. The aim of the present study was to calculate the cumulative risk of LS related cancers in proven *MLH1*, *MSH2 *and *MSH6 *mutation carriers.

**Methods:**

The studypopulation consisted out of 67 proven LS families. Clinical information including mutation status and tumour diagnosis was collected. Cumulative risks were calculated and compared using Kaplan Meier survival analysis.

**Results:**

*MSH6 *mutation carriers, both males and females had the lowest risk for developing CRC at age 70 years, 54% and 30% respectively and the age of onset was delayed by 3-5 years in males. With respect to endometrial carcinoma, female *MSH6 *mutation carriers had the highest risk at age 70 years (61%) compared to *MLH1 *(25%) and *MSH2 *(49%). Also, the age of EC onset was delayed by 5-10 years in comparison with *MLH1 *and *MSH2*.

**Conclusions:**

Although the cumulative lifetime risk of LS related cancer is similar, *MLH1*, *MSH2 *and *MSH6 *mutations seem to cause distinguishable cancer risk profiles. Female *MSH6 *mutation carriers have a lower CRC risk and a higher risk for developing endometrial carcinoma. As a consequence, surveillance colonoscopy starting at age 30 years instead of 20-25 years is more suitable. Also, prophylactic hysterectomy may be more indicated in female *MSH6 *mutation carriers compared to *MLH1 *and *MSH2 *mutation carriers.

## Background

Lynch syndrome (LS), also known as hereditary non-polyposis colorectal cancer, is the most common hereditary colorectal cancer (CRC) syndrome and accounts for 2-5% of all colorectal cancer cases [1]. Germline mutations in any of the four mismatch repair (MMR) genes, *MLH1*[2], *MSH2*[3], *MSH6*[4] and *PMS2*[5], are the underlying cause of LS. Subjects carrying a mutation in one of the MMR genes have a higher risk for developing colorectal cancer, but also for endometrial carcinoma and malignancies of the stomach, small bowel, ovaries, upper uroepithelial tract, biliary tract, skin and brain [6-9].

The colorectal cancer risk in LS is dependent on sex and the MMR gene involved. The reported lifetime risk for colorectal cancer in the literature varies from 28-100% in males and 25-83% in females [7,10-18]. The risk of developing endometrial carcinoma ranges from 30-71% and the risk of other LS-associated cancers is less than 10-15% [9]. Furthermore, some studies have suggested that extracolonic cancers are more often observed in *MSH2 *mutation families compared to *MLH1 *mutation families [13,19]. *MSH6 *mutation families probably have a milder clinical phenotype with a later onset of both CRC and EC and clustering of endometrial carcinoma [17]. The risks in *PMS2 *mutation families are largely unknown. One study reported that PMS2 mutation families have a milder phenotype compared to MLH1 and MSH2 families [20].

Unfortunately, the precise lifetime risk for CRC and endometrial carcinoma may be biased because the families selected in previous studies were mainly selected on basis of clustering of CRC or fulfilment of clinical criteria (Amsterdam II criteria). Furthermore, it was not always clear whether the affected subjects were proven mutation carriers. In addition, most studies have only evaluated lifetime risks for *MLH1 *and *MSH2 *mutations, while studies evaluating *MSH6 *mutation families are sparse. The most efficient way to calculate the lifetime risks of CRC and EC in Lynch syndrome would be to calculate these risks based on a cohort of proven mutation carriers. Therefore, the aim of the present study was to calculate the cumulative lifetime risks for CRC and EC in Lynch syndrome using a cohort of proven *MLH1*, *MSH2 *and *MSH6 *mutation carriers.

## Methods

### Study population

During the period 1994-2007, an MMR gene mutation was detected in 67 families who were counselled at the Department of Clinical Genetics of the Erasmus MC University Medical Center, because of a clinical suspicion for Lynch syndrome. Clinical data of family members including sex, age, mutation status, age at diagnosis of both LS-associated and other cancers were collected. LS-associated cancer included colorectal, endometrial, stomach, ovaries, upper uroepithelial tract, biliary tract, skin and brain cancer. Also, the site of the tumour, age at death and cause of death were collected. With consent of the patients or (in case the patient was deceased) of a close relative the cancer diagnosis was confirmed by pathology and/or medical reports. All pathology and medical reports were reviewed by the first author (DR) in order to confirm the diagnosis. If a subject reported the occurrence of cancer in the family and no pathology or medical report was available, the cancer was excluded from analysis. In addition, data regarding colonoscopic surveillance of affected and unaffected family members were collected.

Only subjects with a proven MMR gene mutation were included in this study.

### Mutation analysis

Mutation analysis was performed by denaturing gradient gel electrophoresis, sequencing and multiplex ligation-dependent probe amplification (MRC-Holland kits P003 and P008). Mutation nomenclature was used according to international guidelines http://www.hgvs.org. A variant was considered a mutation when leading to a predicted truncated protein or based on previously published data. Silent or missense variants which were previously unreported or of unclear status were labelled unclassified variants (UV) and not considered as an MMR gene mutation.

### Statistical analysis

Data were submitted for statistical testing using the Statistical Package for the Social Sciences (SPSS Inc, Chicago, IL), version 12.0.1. Data are given as median and range or as mean with standard deviation when appropriate. The chi square test, Student's t test and log rank test were used to compare differences between *MLH1*, *MSH2 *and *MSH6 *mutation carriers. Penetrance for age was calculated using the Kaplan Meier survival analysis method and included the 67 index cases. In case of multiple or recurrent colorectal carcinoma or endometrial adenocarcinoma, only the first diagnosis of either cancer was included in the analysis. The observation time for the different cancers was from birth until the date of first cancer diagnosis, death, date of hysterectomy (only for the observation time of endometrial carcinoma) or the end of the study (31 December 2007). A p value below .05 was considered statistically significant.

## Results

### Study population

In the 67 families with an MMR gene mutation, 26 (39%) were detected with an *MLH1 *mutation, 20 (30%) with an *MSH2 *mutation and 21 (31%) with an *MSH6 *mutation. Of the 67 families, 46 (69%) met the Amsterdam II criteria. Mutation analysis was performed in 725 subjects (296 men and 429 women) and a mutation was identified in 246 subjects (92 men, 154 women) (Table 1). At the time of mutation analysis the mean age of the 246 mutation carriers was 49 (± 16) years. Of the 246 mutation carriers, 115 (47%) were diagnosed with a Lynch syndrome associated tumour. One hundred and four (42%) mutation carriers already had been diagnosed with a Lynch syndrome associated tumour before mutation analysis was performed. Colorectal cancer was diagnosed in 83 (34%) mutation carriers, including 17 (7%) mutation carriers who developed 2 or more CRCs during their lifetime. Endometrial carcinoma was diagnosed in 37 (24%) of the 154 female mutation carriers, including 13 mutation carriers who also developed CRC during their life. Of the six families with a strong family history of endometrial carcinoma (two or more cases within the family), five (83%) were diagnosed with an *MSH6 *mutation. With respect to the other LS-associated cancers, 19 (8%) mutation carriers developed another LS-associated cancer during their life (Table 1). Seven of these nineteen mutation carriers were also diagnosed with CRC, one mutation carrier also with endometrial carcinoma and four mutation carriers with both CRC and EC. In total, 194 mutation carriers were under colonoscopic surveillance, including 69 subjects who had already been diagnosed with colorectal cancer before mutational testing was performed.

**Table 1:** Study population characteristics

	*MLH1*	*MSH2*	*MSH6*	*Total*
Families	26	20	21	67
Mutation carriers	70	67	109	246
Males (%)	28(40)	28 (42)	36 (33)	92 (37)
				
Subjects with colorectal cancer (%)	36 (51)	21 (31)	26 (24)	83 (34)
Subjects with endometrial carcinoma	7 (10)	9 (13)	21 (19)	37 (15)
				
Subjects with other Lynch associated cancer (%)*				
Ovarian carcinoma	1 (1)	3 (4)	6 (6)	10 (4)
Small bowel cancer	1 (1)	2 (3)	0 (0)	3 (1)
Transitional cell carcinoma	0 (0)	3 (4)	3 (3)	6 (2)

* No histological proven stomach cancers were reported.

One of the 69 mutation carriers had previously been diagnosed with EC and developed CRC while being under colonoscopic surveillance. The other 68 mutation carriers were included in a colonoscopic surveillance program after being diagnosed with colorectal cancer. These 68 subjects were treated surgically (partial colectomy) for colorectal cancer and colonoscopic surveillance of the remaining colon was performed. Of the remaining 125 mutation carriers none developed colorectal cancer and in 23 (18%) adenomatous polyps had been detected and removed. The person-years of follow up was 1414 years and the mean follow up time of the subjects under colonoscopic surveillance was 7 ± 4 years.

### Lifetime risks

**Figure 1:**
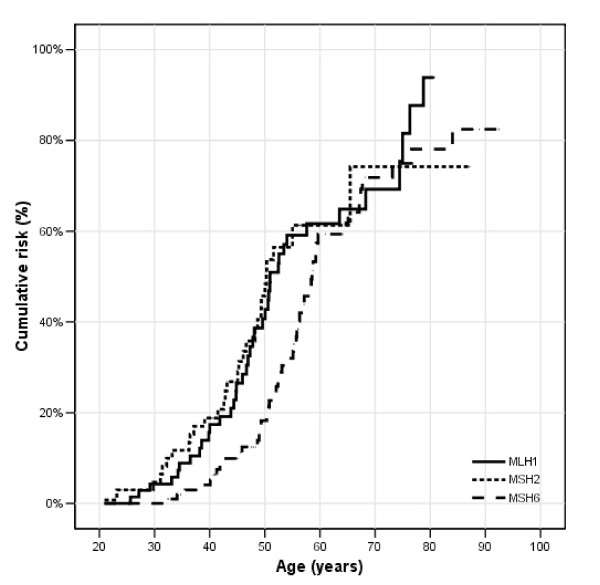
**All Lynch associated cancers (colorectal, endometrial, stomach, ovaries, upper uroepithelial tract, biliary tract, skin and brain cancer): **cumulative risks for *MLH1*, *MSH2 *and *MSH6 *mutation carriers.

**Figure 2:**
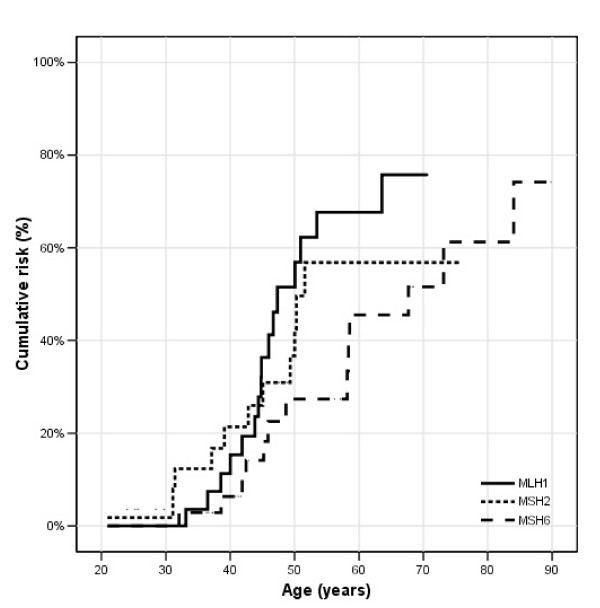
**Colorectal cancer in males; **cumulative risks for *MLH1*, *MSH2 *and *MSH6 *mutation carriers.

**Figure 3:**
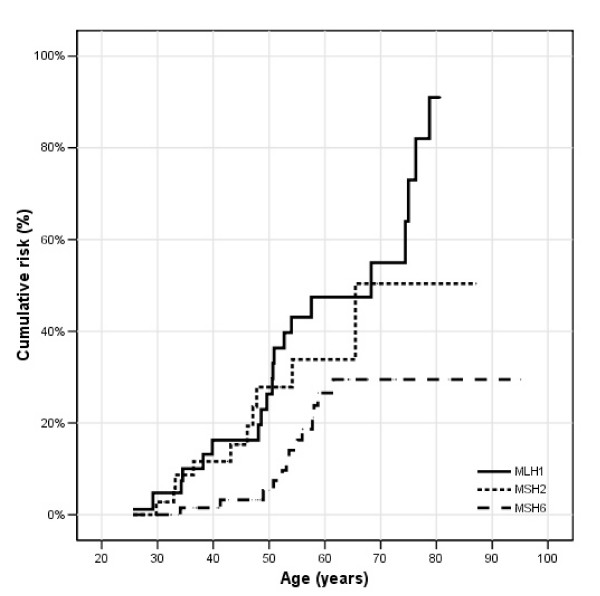
**Colorectal cancer in females; **cumulative risks for *MLH1*, *MSH2 *and *MSH6 *mutation carriers.

**Figure 4:**
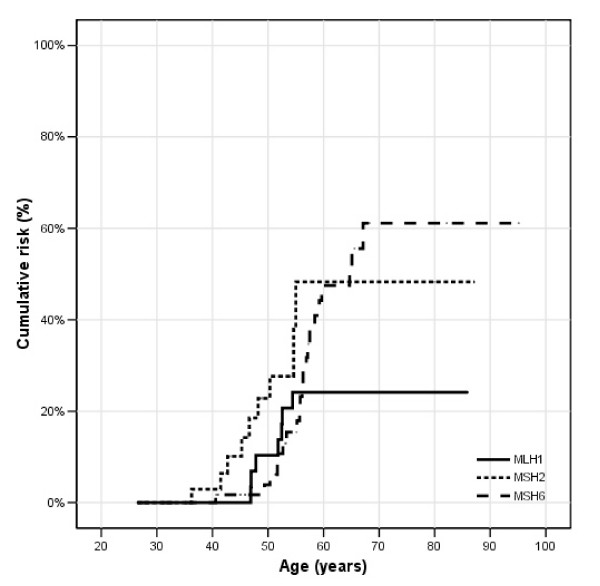
**Endometrial carcinoma in females; **cumulative risks for *MLH1*, *MSH2 *and *MSH6 *mutation carriers.

The respective lifetime risks curves are shown in figure 1, figure 2, figure 3 and figure 4. For all LS-associated tumours, the cumulative risks in both male and female mutation carriers at 70 years was 71% for *MLH1*, 77% for *MSH2 *and 75% for *MSH6 *mutation carriers (Figure 1). Although the cumulative risks at age 70 years were similar for the three different MMR genes, the log rank test showed a significant difference for developing any Lynch syndrome associated cancer between *MSH6*, *MLH1 *and *MSH2 *mutation carriers (p = 0.01). This was due to the fact that before the age of 70 years the risk of developing any Lynch syndrome associated cancer in *MSH6 *carriers was lower compared to *MLH1 *or *MSH2 *mutation carriers (Figure 1).

In Figure 2, the age related cumulative risk for CRC is shown for male *MLH1*, *MSH2 *and *MSH6 *mutation carriers. At age 70 years, the cumulative risk was the highest for *MLH1 *mutation carriers, 78%, while the cumulative risks for *MSH2 *and *MSH6 *mutation carriers were 57% and 54% respectively. There was no significant difference in age related cumulative risk between *MSH6 *mutation carriers (p = 0.05) compared to *MLH1 *and *MSH2 *mutation carriers. However, the highest increase in risk in male *MLH1 *and *MSH2 *mutation carriers was observed between the ages of 40 to 50 years, while the risk in male *MSH6 *mutation carriers mostly increased between the ages of 50 to 60 years. Although the age related risks were not significant different between the three different MMR genes, there was a trend in male *MLH1 *and *MSH2 *mutation carriers to develop CRC at an earlier age than male *MSH6 *mutation carriers. The cumulative risks for CRC in females were lower compared to males, 57% for *MLH1*, 52% for *MSH2 *and 30% for *MSH6 *mutation carriers (Figure 3), with a significantly lower age related cumulative risk in *MSH6 *mutation carriers (p = 0.001) compared to *MLH1 *and *MSH2 *mutation carriers.

For endometrial carcinoma, the highest cumulative risk was observed in the *MSH6 *mutation carriers (61%), while the cumulative risks for *MLH1 *and *MSH2 *mutation carriers were 25% and 49% respectively. However, the log rank test showed no significant difference in age related cumulative risk (p = 0.58) between *MSH6 *mutation carriers compared to *MLH1 *and *MSH2 *mutation carriers.

### Median age of onset

The median age of CRC onset in males was significantly higher in *MSH6 *mutation carriers (48 years; range 32-84 years) compared to *MSH2 *mutation carriers (43 years; range 20-51 years, p = 0.03), but not significantly higher compared to *MLH1 *mutation carriers (45 years; range 33-63 years, p = 0.07) (Table 2). For female mutation carriers, no significant differences in the median age of CRC onset were found when comparing *MSH6 *(53 years; range 34-61 years) with *MLH1 *(50 years; range 25-79 years, p = 0.88) and *MSH2 *(44 years; range 29-82 years, p = 0.28). The median age of EC onset was significant higher in *MSH6 *mutation carriers (56 years; 47-67 years) compared to *MLH1 *mutation carriers (51 years; 46-54 years, p = 0.02) and *MSH2 *mutation carriers (46 years; 36-55 years, p = 0.001). There were no significant differences in the age of onset of other LS-associated cancers between *MLH1 *(53 years; range 52-54 years), *MSH2 *(42 years; range 23-59 years) and *MSH6 *(50 years; range: 35-76) mutation carriers (*MLH1 *vs. *MSH2*, p = 0.41; *MLH1 *vs. *MSH6*, p = 0.76 and *MSH2 *vs. *MSH6*, p = 0.41).

**Table 2:** Median age and range at diagnosis of Lynch syndrome associated cancer

	*MLH1*	*MSH2*	*MSH6*
Colorectal cancer	47 (25-79)	44 (20-82)	53 (32-84)
Endometrial cancer	51 (46-54)	46 (36-55)	56 (47-67)
Ovarian carcinoma	52 (52-52)	47 (45-48)	49 (35-51)
Small bowel cancer	54 (54-54)	36 (23-49)	-
Transitional cell carcinoma	-	58 (32-59)	-

## Discussion

In this study, we evaluated 246 individuals from 67 families with a proven mismatch repair gene mutation to determine the cumulative lifetime risk of developing cancer. Previous studies have shown different lifetime risks for developing CRC in Lynch patients.

One of the first studies evaluating the lifetime risk reported a lifetime risk for CRC at age 75 years of 92% in males and 83% in females [10]. Most later studies reported somewhat similar risks for CRC ranging from 65-100% in males and 30-63% risk in females [7,11-13]. A more recently published study reported the lowest lifetime risk for CRC so far, 27% for males and 22% for females at age 70 years [15]. All these studies only evaluated the risks associated with *MLH1 *and *MSH2 *mutations. Thirty one percent of the families included in our study carried an *MSH6 *mutation. This frequency is higher than previously reported [4,21-23]. Studies evaluating the lifetime risks of cancer amongst *MLH1*, *MSH2 *and *MSH6 *families are sparse. A study evaluating the risk in 20 *MSH6 *families showed that colorectal cancer was less frequent and developed 10 years later in *MSH6 *compared to *MLH1 *and *MSH2*. In addition a significant higher lifetime risk of endometrial carcinoma of 71% in *MSH6 *mutation carriers with a later age of onset (54 years vs. 48 and 49 years for *MLH1 *and *MSH2*) was reported [17]. A German study comparing 27 *MSH6 *mutation families with 156 *MLH1 *and *MSH2 *mutation families confirmed the lower risk and later age of onset of CRC in *MSH6 *families [24]. These results were also confirmed by a recently published British study, but this study only included 11 proven MSH6 mutation carriers [18].

Our study indicates that, however the cumulative risks of cancer at age 70 years in *MLH1*, *MSH2 *and *MSH6 *mutation carriers is similar, each mutated gene has a distinguishable cancer risk profile. In *MSH6 *mutation carriers the risk at age 70 years for developing CRC was the lowest in both male (54%) and female (30%) when compared to carriers of *MLH1 *and *MSH2 *mutations.

Between male *MSH6 *and *MSH2 *mutation carriers also a significant difference in the age of CRC onset (48 vs. 43 years, p = 0.03) was found and there was a trend in higher age of CRC onset between male *MSH6 *and *MLH1 *mutation carriers. For female mutation carriers, no significant differences were found in the mean age of onset of CRC. This can be explained by the fact that female *MLH1 *and *MSH2 *mutation carriers still developed CRC at an older age. The lower risk of CRC onset in female *MSH6 *mutation carriers under the age of 50 years raises the question whether colonoscopic surveillance guidelines in these subjects can be changed. Current guidelines advise to start with biennial colonoscopy surveillance from the age of 20-25 years [25]. In our study population, the youngest affected female *MSH6 *mutation carrier with CRC was 34 years. Our data and the data from previous studies support that colonoscopic surveillance can be started at an age of 30 years in female *MSH6 *mutation carriers [17].

However our numbers are too small to draw definite conclusions, CRC seems to be the predominant cancer in *MLH1 *mutation carriers. In *MSH2 *and *MSH6 *mutation carriers extracolonic cancers appear to contribute more to the similar cumulative lifetime risk of cancer in *MLH1*, *MSH2 *and *MSH6 *mutation carriers. A higher risk of extracolonic-LS-associated cancer was previously reported in *MSH2 *mutation carriers compared to *MLH1 *mutation carriers [13,19]. Unfortunately, the number of extracolonic-LS associated cancer (excluding endometrial carcinoma) in our study population was too low to calculate accurate risk estimates for these cancers. In concordance with other studies [17,26] our study indicates that *MSH6 *carriers have the highest endometrial cancer risk followed by *MSH2 *and *MLH1 *mutation carriers. Also, this risk increases sharply after the age of 50 years. In view of the disputable effect of endometrial carcinoma surveillance [27,28], in female *MSH6 *carriers aged 45 years or above prophylactic hysterectomy may be suggested in order to decrease the risk for developing endometrial carcinoma [29]. In *MSH2 *and *MLH1 *female mutation carriers this option may be more questionable. In *MSH2 *mutation carriers the risk of other extracolonic and extraendometrial cancers may reduce faith in and benefit of risk reducing surgery. In *MLH1 *mutation carriers the risk of endometrial cancer may not outweigh the disadvantages of surgery. In case of surgery for another cause, additional hysterectomy should be considered also in *MLH1 *en *MSH2 *mutation carriers.

A strength of the present study was that the age related risks where calculated using proven mutation carriers. However, the age related risks might be somewhat lower since not all the unaffected individuals from proven mutation families opted for genetic testing and thus the total number of unaffected mutation carriers in the mutation families may be underestimated. In addition, individuals with a higher risk for mutation carriership, i.e. with an affected first degree relative, more often opt for genetic testing [30]. This may also have introduced some bias with respect to the age related risks. Also, we included the index cases in our study population. Index cases give rise to the suspicion of Lynch syndrome and they always have cancer. This may also have resulted in a slightly higher age related risk. On the other hand, the majority (77%) of not affected mutation carriers was under colonoscopy surveillance, which likely has influenced the age related risks for developing invasive CRC, since colonoscopy surveillance in Lynch syndrome patients is effective in reducing the incidence and mortality of CRC [31]. A limitation of our study was that our study population was not very large (n = 246), and the number of male carriers was 92. This could explain why we did not find a significant difference in both the mean age of CRC onset and the age related risk between male *MLH1*, *MSH2 *and *MSH6 *mutation carriers.

In conclusion, the present study indicates that, although the cumulative risks at age 70 years of LS related cancer in *MLH1*, *MSH2 *and *MSH6 *mutation carriers are similar, each mutated gene has a distinguishable cancer risk profile. It underlines that female *MSH6 *mutation carriers have a distinct clinical phenotype with a lower CRC risk and a higher risk for developing endometrial carcinoma. Starting with biennial colonoscopic surveillance at an age of 30 years instead of an age of 20-25 years in female *MSH6 *mutation carriers is more suitable. Moreover, in female *MSH6 *mutation carriers prophylactic hysterectomy may be considered from an age of 45 years.

## Conclusions

The present study indicates that each mutated MMR gene has a distinguishable cancer risk profile. Female *MSH6 *mutation carriers have a lower CRC risk and a higher risk for developing endometrial carcinoma. Starting with biennial colonoscopic surveillance at an age of 30 years in female *MSH6 *mutation carriers is more suitable and prophylactic hysterectomy may be considered from an age of 45 years.

## Abbreviations

CRC: colorectal cancer; EC: endometrial cancer; LS: Lynch syndrome; MMR: mismatch repair; UV: unclassified variant.

## Competing interests

The authors declare that they have no competing interests.

## Authors' contributions

DR participated in the data collection, performed the statistical analyses and helped to draft the manuscript. AW conceived of the study and participated in the data collection. ML helped to draft the manuscript. DD participated in the data collection. CT participated in the data collection. ES participated in the design of the study and assisted in the statistical analysis. EK helped to draft the manuscript. All authors read and approved the final manuscript.
